# Dual inhibition of COX‐2 and sEH improves cognitive function in AD associated with enhanced cerebral vascular function

**DOI:** 10.1002/alz70855_096822

**Published:** 2025-12-23

**Authors:** Gilbert Charles Morgan, Chengyun Tang, Andrew Gregory, Fan Fan

**Affiliations:** ^1^ Augusta University, Augusta, GA, USA

## Abstract

**Background:**

Genetic evidence ties *EPHX2* (encoding sEH) and *PTGS2* (encoding COX‐2) to AD/ADRD. Both sEH and COX‐2 were elevated in AD/ADRD patients and animal models, affecting pathways related to neurodegeneration, glial activation, vascular function, and inflammation. We previously showed that 3‐month sEH inhibition with TPPU (1 mg/kg/day) improved cerebral hemodynamics and cognition in AD/ADRD. This study evaluates the effects of a novel dual COX‐2 and sEH inhibitor on cerebral vascular function and cognition in AD.

**Methods:**

TgF344‐AD rats were orally administered PTUPB. Cognitive function was evaluated using the Novel Object Recognition test. Cerebral vascular function was assessed through pressure myography to measure the myogenic responses of the middle cerebral arteries and penetrating arterioles. Additionally, a transcriptomic profile was generated from bulk RNA‐seq analysis of primary VSMCs isolated from AD rats treated with either PTUPB or vehicle.

**Results:**

Body weights, plasma glucose, and HbA1c levels were unaltered in vehicle and drug‐treated AD rats. Dual inhibition of COX‐2 and sEH improved recognition memory in AD rats associated with enhanced myogenic responses of the freshly isolated middle cerebral arteries and penetrating arterioles. Transcriptomic analysis of primary cerebral vascular smooth muscle cells from AD rats treated with PTUPB revealed enhanced pathways related to cell contraction, alongside decreased oxidative stress and inflammation.

**Conclusions:**

These findings provide novel evidence that dual inhibition of COX‐2 and sEH can reverse cerebrovascular dysfunction and cognitive impairments in AD, demonstrating greater potency than sEH inhibition alone. Our study presents a promising avenue for therapeutic development.

**Financial Disclosure**: None.

**Funding Resources**: This study was supported by grants AG079336, and AG057842, from the National Institutes of Health, TRIBA Faculty Startup Fund from Augusta University, and 25PRE1365157 from the American Heart Association.

